# NFAT1 enhances the effects of tumor-associated macrophages on promoting malignant melanoma growth and metastasis

**DOI:** 10.1042/BSR20181604

**Published:** 2018-12-18

**Authors:** Hao Liu, Liping Yang, Min Qi, Jianglin Zhang

**Affiliations:** 1 Department of Dermatology, Xiangya Hospital, Central South University, Changsha, Hunan Province 410008, P.R. China; 2 College of Life Sciences, Hunan Normal University, Changsha, Hunan Province 410006, P.R. China; 3 Department of Plastic and Cosmetic Surgery, Xiangya Hospital, Central South University, Changsha, Hunan Province 410008, P.R. China

**Keywords:** melanoma, NFAT1, tumor-associated macrophages

## Abstract

Tumor-associated macrophages (TAMs) play substantial roles in tumor growth, invasion, and metastasis. Nuclear factor of activated T cell (NFAT1) has been shown to promote melanoma growth and metastasis *in vivo*. We herein aim to investigate whether NFAT1 is capable to promote melanoma growth and metastasis by influencing TAM properties. Melanoma-conditioned TAMs were obtained from human monocytes after incubation with conditioned medium from A375 cell culture. The phenotype of the macrophages was detected. Cell proliferation, migration, and invasion were evaluated. Human malignant melanoma tissues exhibited increased CD68^+^-macrophage infiltration and NFAT1 expression compared with the normal pigmented nevus tissues. Melanoma-conditioned TAMs displayed M2-like phenotype. Melanoma-conditioned TAMs also promoted proliferation, migration, and invasion of human malignant melanoma cell lines A375 and WM451. Furthermore, NFAT1 expression in TAMs was significantly increased compared with the M0 group. NFAT1 overexpression significantly strengthened the melanoma-conditioned TAM-mediated promotion of cell migration and invasion in A375 and WM451 cells, whereas NFAT1 knockdown exerted the opposite effects. Moreover, NFAT1 overexpression in melanoma-conditioned TAMs promoted CD68^+^-macrophage infiltration, tumor growth, and metastasis *in vivo*. NFAT1 may play a critical role in enhancing the TAM-mediated promotion of growth and metastasis in malignant melanoma.

## Background

Melanoma is the most aggressive and the deadliest form of skin cancer [1]. Current therapeutic approaches include surgical resection, immunotherapy, chemotherapy, biochemotherapy, photodynamic therapy, and targetted therapy [2–4]. Despite rapid progress in the treatment, malignant melanoma still presents a major clinical challenge.

The melanoma progression and pathogenesis strongly depends on the fine-tuned interactions between melanoma cancer cells and the various stromal cells in the host [5]. Amongst them, tumor-associated macrophages (TAMs) have been shown to play substantial roles in tumor growth, invasion, and metastasis [6–9]. Convincing evidence has highlighted the association of increased TAMs infiltration with poor prognosis and worse pathological characteristics in diverse cancers, including colon cancer, breast cancer, bladder cancer, and also melanoma [10–14]. A spectrum of TAM phenotypes have been shown to exist in tumors; however, two opposing phenotypes, named classically activated macrophage (M1)-like and alternatively activated macrophages (M2)-like phenotypes, have been demonstrated to be related to anti- and pro-tumoral functions, respectively [3,15]. TAMs generally acquire M2-like properties [16,17].

Nuclear factor of activated T cell (NFAT1, NFATC2) is a transcription factor that binds and positively regulates the expression of interleukin (IL)-2 (IL-2) during T-cell activation [18]. Previous studies have suggested that NFAT1 play important roles in both innate and adaptive immune responses [18]. Furthermore, NFAT1 has been implicated in various tumor progression events such as tumor cell migration, invasion, survival, and apoptosis [19]. A recent study has demonstrated that NFAT1 promotes melanoma tumor growth and metastasis via direct regulation of IL-8 and matrix metalloproteinase (MMP)-3 (MMP-3) [18]. In this study, increased NFAT1 expression has been observed in M2 macrophages based on Gene Expression Omnibus (GEO) database (www.ncbi.nlm.nih.gov/geo/), a public functional genomics data repository. However, NFAT1 expression in TAMs and its regulation of TAM properties remain unclear.

Accordingly, the present study aimed to investigate whether NFAT1 is capable of promoting melanoma growth and metastasis by influencing TAM properties.

## Materials and methods

### Immunohistochemistry

Immunohistochemistry (IHC) was performed to detect CD68 and NFAT1 levels in human malignant melanoma tissues and normal pigmented nevus tissues. Briefly, consecutive paraffin sections (4-μm-thick) were deparaffinized and rehydrated, followed by a block of endogenous peroxidase activity and antigen retrieval. Sections were incubated with a primary mouse anti-human CD68 (1:100; Abcam, Cambridge, MA, U.S.A.), mouse anti-human NFAT1 (1:125; Abcam) at 37°C for 1 h and then with goat anti-mouse IgG H&L (HRP) (Abcam) at room temperature for 30 min. The sections were stained with diaminobenzidine (DAB), counterstained in Hematoxylin solution, dehydrated, and then embedded in paraffin. The yellowish-brown staining indicates a positive signal. The sections were observed under an Olympus BH-2 light microscope (Olympus, Tokyo, Japan).

### Isolation and culture of human peripheral blood macrophages

The study was approved by the Ethics Committee of Xiangya Hospital, Central South University. Human peripheral blood macrophages were isolated and cultured, and M0, M1, M2 macrophages were induced respectively as previously described [20]. Briefly, blood monocytes were isolated from healthy donor buffy coats. Peripheral blood mononuclear cells (PBMCs) were isolated using a Ficoll (Solarbio Life Sciences, Beijing, China) density gradient. Monocytes were purified with anti-CD14 paramagnetic beads (Miltenyi Biotec, Auburn, CA). CD14^+^ monocytes (5 × 10^5^ cells/ml) were cultured with RPMI 1640 (Sigma, St. Louis, MO, U.S.A.), supplemented with 10% FBS (Sigma), and macrophage colony-stimulating factor (M-CSF, 50 ng/ml; Sigma) for 7 days. To obtain M0 cells, CD14^+^ monocytes were treated with serum-free medium for 48 h. To polarize M1 macrophages, cells were stimulated overnight with lipopolysaccharides (LPS, 100 ng/ml; Sigma) and IFN-γ (100 ng/ml; Sigma). To polarize M2 macrophages, cells were stimulated overnight with IL-4 (20 ng/ml; Sigma). TAMs of melanoma were obtained by culturing monocytes isolated from PBMCs for 7 days in RPMI 1640 containing 10% FBS with 50% of conditioned medium from A375 cells. Conditioned medium was obtained from untreated A375 cells.

### Western blot

Cells were lysed with RIPA lysis buffer (Santa Cruz Biotechnology, Dallas, TX, U.S.A.). Equal amount of protein from cell lysates was separated by SDS/PAGE (10% gels) and transferred to PVDF membranes (Millipore Corp., Billerica, MA, U.S.A.). After being blocked with 5% fat-free milk, the membranes were incubated with the following primary antibodies: CD163 (1:1000; Abcam) and GAPDH (1:10000; Abcam; the loading control) at room temperature for 3 h, and then incubated with mouse anti-rabbit secondary antibody (1:10000; Abcam). Blots were developed using an ECL kit (Pierce Biotechnology, IL) and band intensity was quantitated with Quantity One software.

### Detection of cytokine levels

Levels of cytokines including TNF-α, IL-1β, TGF-β, and IL-10 were measured using their commercial ELISA kits (R&D Systems, Minneapolis, MN, U.S.A.), according to the manufacturer’s instructions.

### Cell culture

Human malignant melanoma A375 cells were purchased from the Cell Bank of Chinese Academy of Sciences (Shanghai, China). Human malignant melanoma WM451 cells were purchased from Yansheng Biotechnology Co. (Shanghai, China). Cells were cultured at 37°C in a humidified atmosphere with 5% CO_2_ in Dulbecco’s modified Eagle’s medium (DMEM, Gibco, Thermo Fisher Scientific, Inc., Waltham, MA, U.S.A.), supplemented with 10% FBS, 100 IU/ml streptomycin, and 100 IU/ml penicillin.

### Cell counting kit-8 assay

Cell viability was tested by Cell Counting Kit-8 (CCK-8, Beyotime, Shanghai, China) assay as previously described [21], with some alterations. Cells were seeded into 96-well plates at a density of 4000 cells/well and incubated for 24 h. Afterward, 10 μl of CCK-8 solution was added to each well, followed by incubation for 2 h. The absorbance was determined by a microplate reader (Molecular Devices, Sunnyvale, CA, U.S.A.) at a wavelength of 450 nm (OD value). Columns represented the mean percentage of OD values relative to the negative control. Each experiment was performed four times.

### Transwell migration and invasion assay

Transwell migration and invasion assay were performed as previously described [22]. The migratory cells were fixed using 4% paraformaldehyde and were stained using 0.1% Crystal Violet solution. Then, the number of migratory cells was counted. The same procedures were followed for the invasion assay, except diluted Matrigel (BD Biosciences, San Jose, CA, U.S.A.) were precoated on the upper well of the transwell chamber and incubated it for 1 h at 37°C.

### RNA extraction and real-time reverse transcription PCR

Total RNA was extracted from liver tissues or primary hepatocytes using TRIzol reagent (Invitrogen, Thermo Fisher Scientific, Inc., Waltham, MA, U.S.A.) according to the manufacturer’s instructions. Total RNA was reverse transcribed using the iScript kit (Bio-Rad, Hercules, CA, U.S.A.), and real-time PCRs were performed on the CFX96™ real-time PCR detection system (Bio-Rad) using Power SYBR Green RT-PCR reagents. PCRs were performed using a TaqMan Master Mix (Applied Biosystems, Foster City, CA, U.S.A.). Data were presented as relative quantitation based on the calculation of 2^−ΔΔ*C*^_t_.

### Plasmids construction and cell transfection

To overexpress NFAT1, the full-length NFAT1 cDNA fragment was cloned into pcDNA 3.1, generating pcDNA3.1-NFAT1. To knock down NFAT1, scramble siRNA and NFAT1 siRNA (si-NFAT1) were conducted. A375 and WM451 cells were then transfected with the respective plasmids using Lipofectamine 2000 (Invitrogen) according to the manufacturer’s instructions. Relative NFAT1 expression was assessed by qRT-PCR to determine the overexpression and knockdown efficiency in A375 and WM451 cells.

### Lentivirus preparation and transduction

Lentivirus-expressing NFAT1 (NFAT1 OE) and empty expression lentiviral vectors (control) were constructed by GenePharma (Shanghai, China). TAMs were transduced with lentivirus-NFAT1 OE as previously described [23].

### Animals, tumor growth, and metastasis

Thirty-two female athymic BALB/c nude mice were (8 weeks old) were obtained from Charles River Laboratories (Beijing, China). All animals were housed under specific pathogen-free conditions, controlled temperature (25 ± 1°C) and humidity (50%), with 12-h light/dark cycles and free access to food and water. All animal experiments were approved by the Ethics Committees of the Xiangya Hospital, Central South University.

For tumor growth experiments, tumors were produced by subcutaneously injecting a mixture of A375 human melanoma cells and lentivirus-expressing NFAT1-transduced TAMs (1 × 10^6^ cell/mouse; 1:1) into the right flank of each mouse (*n*=8/group). Tumor size was recorded every 5 days with a caliper and calculated as a × b^2^/2 mm^3^ (a, long diameter; b, short diameter). Thirty-five days later, mice were killed, and the tumors were separated and processed for IHC to detect CD68 expression. For lung metastasis experiments, mice were injected with 1 × 10^6^ cells via lateral tail vein injections as previously described [24]. *n*=8 for each group in metastasis experiments.

### Statistical analysis

All data were presented as the mean ± S.D. of three independent experiments. All statistical analyses were performed using the SPSS 16.0 (SPSS, Inc., Chicago, IL, U.S.A.). One-way ANOVA was used for comparison. Statistical significance was set at *P*<0.05.

## Results

### Increased CD68 and NFAT1 expression in malignant melanoma tissues

**Figure 1:**
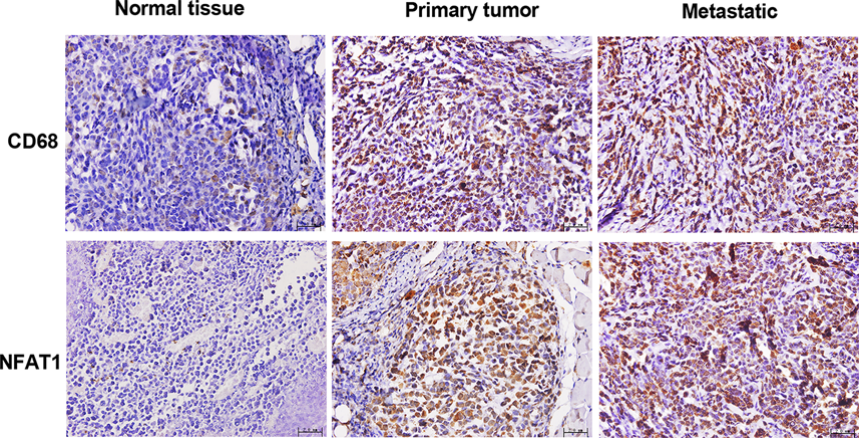
Increased CD68 and NFAT1 expression in malignant melanoma tissues Human primary and metastatic melanoma tissues were collected, and normal pigmented nevus tissues were used as controls. IHC was performed to detect CD68 for labeling TAM and NFAT1 levels. Scale bar = 2 µm.

As shown in Figure 1, human primary and metastatic melanoma specimens exhibited increased infiltration of macrophages, as indicated by increased CD68-positive (yellowish-brown) granules in the cytoplasm and/or cell membrane compared with the normal pigmented nevus tissues. Furthermore, NFAT1 expression was also elevated in human primary and metastatic malignant melanoma specimens compared with the normal pigmented nevus tissues. These data indicated the potential role of TAM and NFAT1 in malignant melanoma.

### Melanoma-conditioned TAMs possessed M2-like phenotype

**Figure 2:**
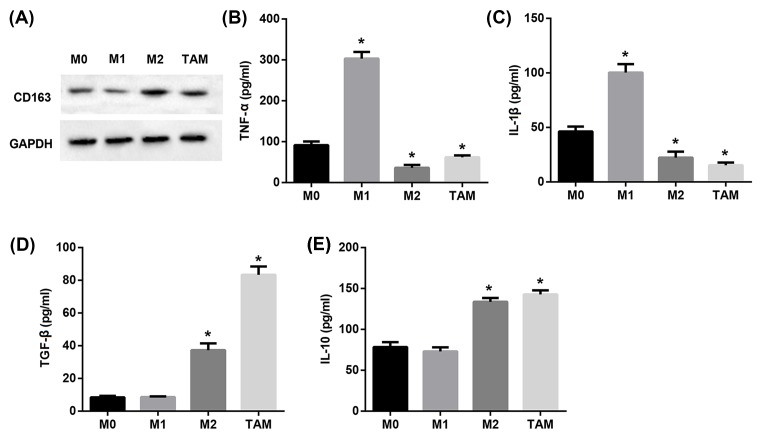
Characterization of melanoma-conditioned TAMs (**A**) Western blot was performed to examine the protein expression of CD163. ELISA was used to examine levels of cytokines including TNF-α (**B**), IL-1β (**C**), TGF-β (**D**), and IL-10 (**E**) secreted in the culture medium. *P<0.05 compared with the M0 group.

We next incubated human blood monocytes with culture medium from A375 cells. Seven days later, the phenotype of the macrophages was detected to investigate whether melanoma tumor microenvironment exposure affected monocyte differentiation. Western blot analysis revealed that the protein expression of M2 marker CD163 in M1 group was significantly lower than that in the M0 group (Figure 2A). In contrast, CD163 protein expression in M2 and TAM group was notably higher than that in the M0 group (Figure 2A). Moreover, a TNF-α ^low^, IL-1β ^low^, TGF-β ^high^, and IL-10 ^high^ phenotype was observed in TAMs (Figure 2B–E). These results suggested that melanoma-conditioned TAMs displayed M2-like phenotype.

### Melanoma-conditioned TAMs promote proliferation, migration, and invasion of A375 and WM451 cells *in vitro*

**Figure 3:**
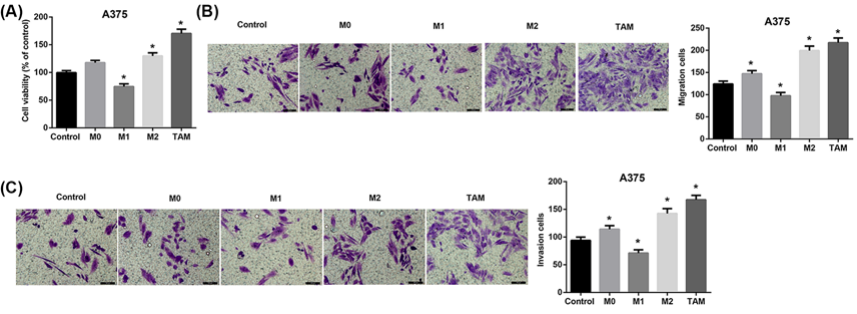
Effect of melanoma-conditioned TAMs on proliferation, migration, and invasion of A375 cells *in vitro* A375 cells were incubated with culture medium of M0, M1, M2, and TAMs cells for 48 h. (**A**) Cell viability of A375 cells was detected using CCK-8 assay. Cell migration (**B**) and invasion (**C**) of A375 cells were evaluated using Transwell assays. Scale bar = 100 µm. *P<0.05 compared with the control group.

**Figure 4:**
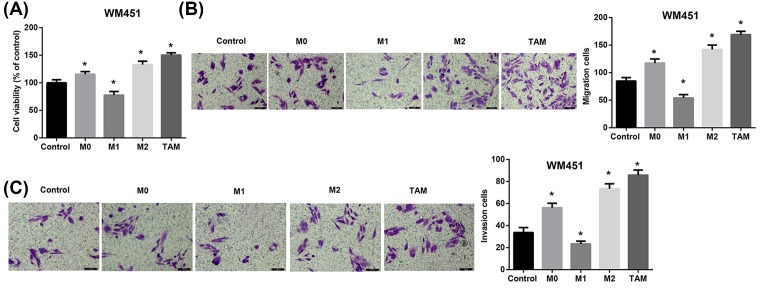
Effect of melanoma-conditioned TAMs on proliferation, migration, and invasion of WM451 cells *in vitro* WM451 cells were incubated with culture medium of M0, M1, M2, and TAMs cells for 48 h. (**A**) Cell viability of WM451 cells was detected using CCK-8 assay. Cell migration (**B**) and invasion (**C**) of WM451 cells were evaluated using Transwell assays. Scale bar = 100 µm. *P<0.05 compared with the control group.

Subsequently, we explored whether these TAMs had the M2-like pro-tumoral effects on A375 and WM451 cells. To address this, we incubated A375 and WM451 cells with culture medium of M0, M1, M2, and TAM cells or RPMI 1640 medium (control) for 48 h. Compared with the control cells, A375 cells incubated with M2 macrophages and TAMs exhibited a significant promotion in cell proliferation (Figure 3A), migration (Figure 3B), and invasion (Figure 3C). Similar results were shown in WM451 cells (Figure 4A–C).

### Melanoma-conditioned TAMs overexpressing or silencing NFAT1 regulate migration and invasion of A375 and WM451 cells

**Figure 5:**
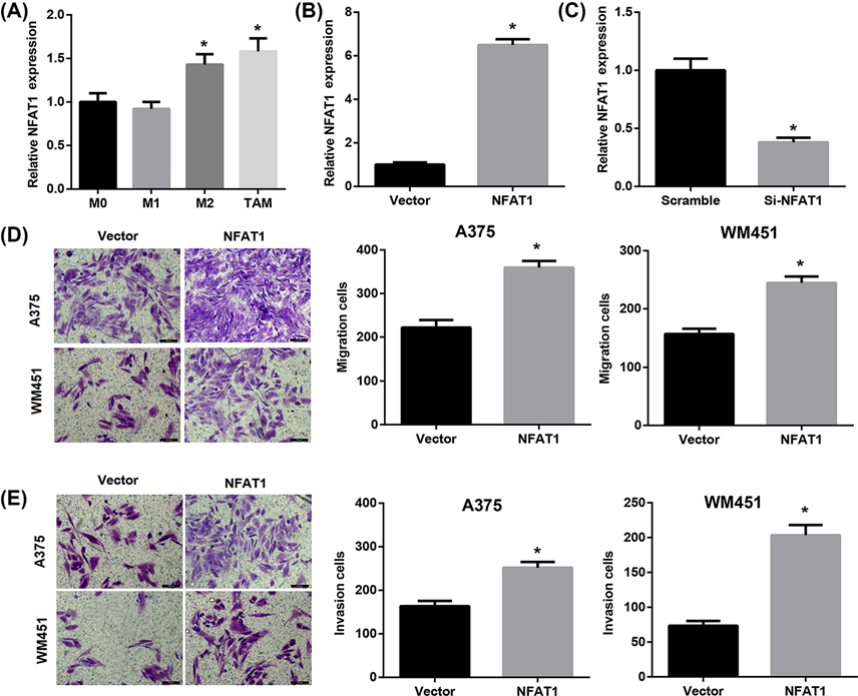
Melanoma-conditioned TAMs that were transfected with pcDNA3.1-NFAT1 regulate migration and invasion of A375 and WM451 cells (**A**) Relative NFAT1 expression in M0, M1, M2, and TAMs was detected by qRT-PCR. *P<0.05 compared with the M0 group. A375 and WM451 cells were incubated with culture medium from TAMs that had been transfected with pcDNA3.1-NFAT1, si-NFAT1, or the corresponding controls. The overexpression (**B**) and knockdown efficiency (**C**) of NFAT1 was confirmed by qRT-PCR. *P<0.05 compared with the vector or scramble group. A375 and WM451 cell migration (**D**) and invasion (**E**) were evaluated using Transwell assays. Scale bar = 100 µm. *P<0.05 compared with the vector group.

**Figure 6:**
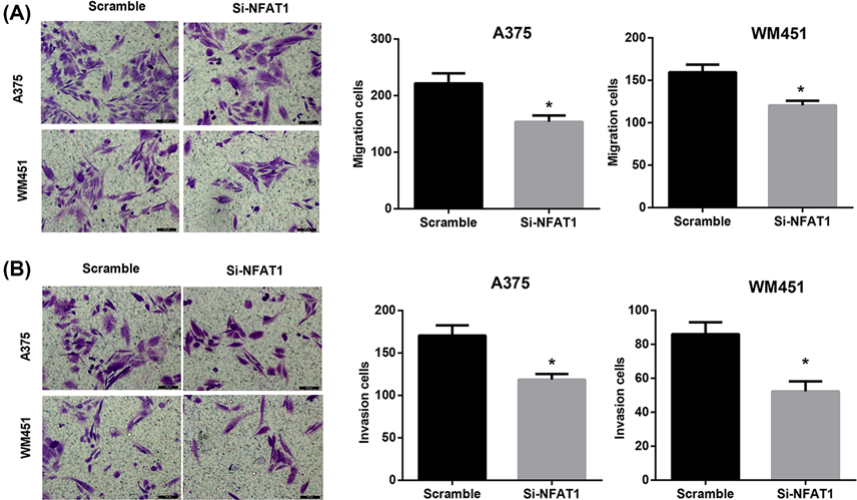
Melanoma-conditioned TAMs that were transfected with si-NFAT1 regulate migration and invasion of A375 and WM451 cells A375 and WM451 cells were cultured with conditioned medium from TAMs that had been transfected with si-NFAT1 or the scramble control for 48 h. Cell migration (**A**) and invasion (**B**) of A375 and WM451 cells were evaluated using Transwell assays. Scale bar = 100 µm. *P<0.05 compared with the scramble group.

Next, NFAT1 expression in M0, M1, M2, and TAMs was detected. Data demonstrated that relative NFAT1 expression was significantly increased in M2 and TAMs compared with the M0 group (Figure 5A). To clarify the effects of NFAT1 expression on A375 and WM451 cell growth in TAMs, we incubated A375 and WM451 cells with culture medium from TAMs that had been transfected with pcDNA3.1-NFAT1, si-NFAT1, or the corresponding controls. The overexpression and knockdown efficiency of NFAT1 was confirmed by qRT-PCR (Figure 5B,C). The results showed that the capacity of melanoma-conditioned TAMs to promote cell migration and invasion of A375 and WM451 cells was significantly enhanced by NFAT1 overexpression (Figure 5D,E) but suppressed by NFAT1 knockdown (Figure 6A,B) compared with their corresponding control. These data indicated the important role of NFAT1 expression in melanoma-conditioned TAMs in regulating migration and invasion of A375 and WM451 cells.

### NFAT1 overexpression in melanoma-conditioned TAMs promotes tumor growth and metastasis

**Figure 7:**
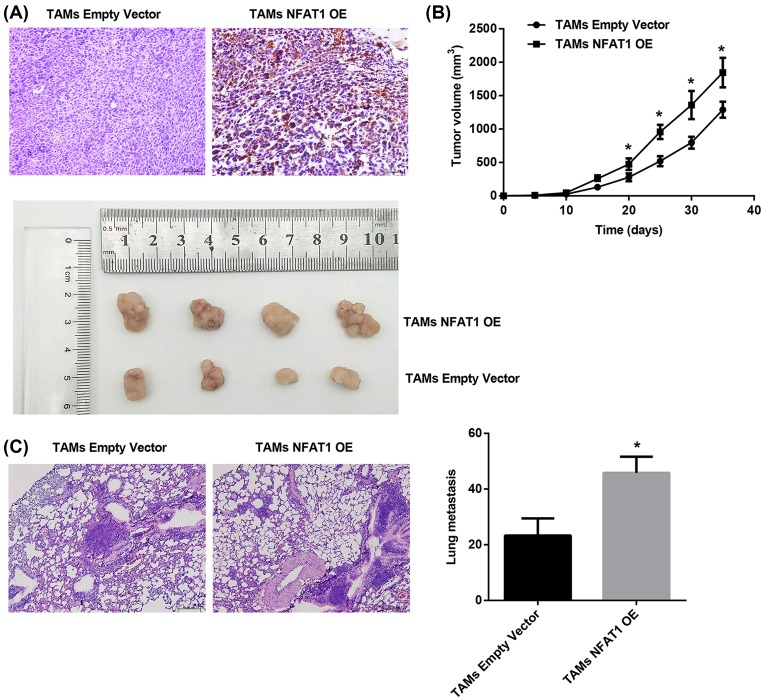
Effect of NFAT1 overexpression in melanoma-conditioned TAMs on tumor growth and metastasis *in vivo* A375 and lentivirus-expressing NFAT1-transduced TAMs (1 × 10^6^ cell/mouse; 1:1) were injected into BALB/c nude mice. Thirty-five days later, mice were killed and the tumors were separated. (**A**) IHC staining for CD68 from *in vivo* tumors. Scale bar = 2 µm. (**B**) Tumor volumes monitored every 5 days. Representative images showing tumor size. (**C**) Representative images showing metastatic nodules in the lung and quantitation of lung metatasis. Scale bar = 5 µm. *n*=8 for each group. *P<0.05 compared with the TAMs empty vector group.

Silencing of NFAT1 expression in metastatic melanoma cells suppressed tumor growth and metastatic potentials *in vivo* [25]. To further explore whether NFAT1 silencing led to a decrease in melanoma cell tumorigenicity and metastatic potential was associated with TAMs, here we developed xenografts with an injection of a mixture of A375 human melanoma cells and lentivirus-expressing NFAT1-transduced TAMs. IHC staining demonstrated increased macrophage infiltration after NFAT1 overexpression, as indicated by an increased number of CD68-positive macrophages in the TAMs NFAT1 OE group compared with the TAMs empty vector group (Figure 7A). Furthermore, a significant increase in both tumor growth and the number of lung metastases was observed in the TAMs NFAT1 OE group compared with the TAMs empty vector group (Figure 7B,C). These data suggested that the critical role of NFAT1 expression in melanoma-conditioned TAMs in regulating melanoma growth and lung metastasis *in vivo*.

## Discussion

In response to various microenvironmental stimuli, macrophages can be polarized to achieve several functional phenotypes [26]. It has been well-established that M1 macrophages are pro-inflammatory, pro-immunity, and anti-tumor, whereas M2 macrophages are anti-inflammatory, immune suppressive, proangiogenic, and pro-tumor [27]. In general, TAMs acquire an M2-like phenotype that is relevant for their participation in tumor progression [16,17]. A previous study revealed that the main type of tumor-infiltrating macrophage in uveal melanoma was the M2 type [28]. This is in-line with our finding that melanoma-conditioned TAMs displayed M2-like phenotype, including increased CD163 protein expression and TNF-α ^low^, IL-1β ^low^, TGF-β ^high^ and IL-10 ^high^ phenotype.

TAMs, especially M2-like TAMs, have been shown to exert important tumor-supportive roles. Increased numbers of TAMs positively correlate with poor prognosis, greater invasiveness, and metastasis in melanoma [29,30]. Studies also indicated that melanoma-associated macrophages drive melanoma growth [30,31]. Consistent with the pro-tumoral role of TAMs in melanoma, our results showed that M2-like melanoma-conditioned TAMs promoted proliferation, migration, and invasion of human malignant melanoma cell lines A375 and WM451.

TAMs are a critical component of tumor microenvironments, which affect tumor growth, angiogenesis, metastasis, immune suppression, and chemoresistance [32]. Macrophages infiltrate into the tumor microenvironment and secrete certain factors to facilitate tumor proliferation and migration. The number of tumor-infiltrating CD68^+^ macrophages contributes to the prognosis in uveal melanoma [33]. Furthermore, Kale et al. [12] emphasized the potential role of TAMs in modulating tumor microenvironment via secretion of osteopontin (OPN), prostaglandin E2 (PGE2), and MMP-9, which trigger angiogenesis and melanoma growth. Studies have also demonstrated the involvement of NFAT1 in the tumor microenvironment [18,34,35]. In this study, we observed that human malignant melanoma tissues exhibited increased infiltration of CD68^+^ macrophages and NFAT1 expression compared with the normal pigmented nevus tissues. These data indicated the potential association between TAMs and NFAT1.

NFAT1 is implicated in cancer proliferation, invasion, apoptosis, angiogenesis, and lymphangiogenesis [34]. For example, NFAT1 is overexpressed in glioblastoma multiforme (GBM) and contributed to the invasive potential of GBM cells [19]. Augmented expression of NFAT1 was also detected in lung cancer tissues and correlated with poor prognosis of patients with lung cancer [36]. Also in human melanoma, NFAT1 was constitutively expressed both *in vitro* and *in vivo* and NFAT1 silencing promotes melanoma cell apoptosis [37]. Data from a recent publication have also demonstrated that NFAT1 promotes melanoma tumor growth and metastasis via direct regulation of IL-8 and MMP-3 [18]. Human malignant melanoma tissues exhibited increased infiltration of CD68^+^ TAMs and NFAT1 expression compared with the normal pigmented nevus tissues.

Similar to increased NFAT1 expression in M2 macrophages based on GEO database, our findings revealed that NFAT1 expression in M2-like TAMs was significantly increased compared with the M0 group. In the present study, to investigate whether NFAT1 is capable to promote melanoma growth and metastasis by influencing TAM properties, A375 and WM451 cells were cultured with conditioned medium from TAMs that had been transfected with pcDNA3.1-NFAT1, si-NFAT1 for 48 h. Our results revealed that NFAT1 overexpression significantly strengthened the promotion of cell migration and invasion in A375 and WM451 cells that were mediated by melanoma-conditioned TAMs, whereas NFAT1 knockdown exerted opposite effects. Moreover, NFAT1 overexpression in melanoma-conditioned TAMs promoted CD68^+^-macrophage infiltration, tumor growth, and metastasis *in vivo*. These data suggested that increased NFAT1 expression in TAMs was involved in the TAMs-mediated facilitation of tumor growth and metastasis. Our present study further elucidated the role of NFAT1 in melanoma progression.

## Conclusion

In conclusion, NFAT1 may have an important role in enhancing the TAM-mediated promotion of growth and metastasis in malignant melanoma. NFAT1 can act as a promising modulator for TAMs’ functions and may, therefore, serve as a promising therapeutic target for melanoma treatment.

## Abbreviations

CCK-8: cell counting kit-8
GAPDH: glyceraldehyde-3-phosphate dehydrogenase
GBM: glioblastoma multiforme
GEO: Gene Expression Omnibus
HRP: horseradish peroxidase
IFN-γ: interferon-γ
IHC: immunohistochemistry
IL: interleukin
MMP: matrix metalloproteinase
NFAT1: nuclear factor of activated T cell
OD: optical density
PBMC: peripheral blood mononuclear cell
qRT-PCR: quantitative real-time polymerase chain reaction
RIPA: radioimmunoprecipitation assay
RPMI 1640: Roswell Park Memorial Institute-1640
TAM: tumor-associated macrophage
TGF-β: transforming growth factor-β
TNF-α: tumor necrosis factor-α
